# *IRF6* and *FGF1* polymorphisms in non-syndromic cleft lip with or without cleft palate in the Polish population

**DOI:** 10.1515/med-2023-0677

**Published:** 2023-04-01

**Authors:** Alicja Zawiślak, Krzysztof Woźniak, Beata Kawala, Satish Gupta, Anna Znamirowska-Bajowska, Joanna Janiszewska-Olszowska, Jan Lubiński, José Luis Calvo-Guirado, Katarzyna Grocholewicz, Anna Jakubowska

**Affiliations:** Department of Maxillofacial Orthopaedics and Orthodontics, Institute of Mother and Child, 01-211 Warsaw, Poland; Department of Interdisciplinary Dentistry, Pomeranian Medical University, 70-111 Szczecin, Poland; Department of Orthodontics, Pomeranian Medical University, 70-111 Szczecin, Poland; Department of Dentofacial Orthopaedics and Orthodontics, Wrocław Medical University, 50-425 Wrocław, Poland; Hereditary Cancer Center, Department of Genetics and Pathology, Pomeranian Medical University, 70-111 Szczecin, Poland; Department of Oral Surgery and Implant Dentistry, Faculty of Health Sciences, Universidad Católica de Murcia, UCAM, 30107, Murcia, Spain

**Keywords:** birth defect, cleft lip, cleft palate, genetic variation, single nucleotide polymorphism, *IRF6*, *FGF1*

## Abstract

Non-syndromic cleft lip with or without cleft palate (NSCL/P) is the most common developmental defect that significantly affects the morphology and function of the stomatognathic system in children. The etiology of these birth defects is multifactorial, and single nucleotide polymorphisms (SNPs) in *IRF6* and *FGF1* have been associated with NSCL/P. This study aimed to evaluate whether SNPs in *IRF6*, namely rs2013162, rs642961, rs2235373, and rs34010 in *FGF1*, are associated with NSCL/P occurrence in the Polish population. The study included 627 participants: 209 children with NSCL/P and 418 healthy controls. DNA was isolated from saliva in the study group and from umbilical cord blood in controls. Genotyping of polymorphisms was performed using quantitative PCR. There was no statistically significant association of *IRF6* gene variants with NSCL/P occurrence, although for rs2013162, AA genotype, odds ratio (OR) = 1.16 and for AC genotype, OR = 0.83; for rs642961, AA genotype, OR = 0.84 and for AG genotype, OR = 1.41; and for rs2235373, AA genotype, OR = 0.79 and for AG, OR = 0.85. In the instance of rs34010 polymorphism in *FGF1*, the presence of the AA genotype was statistically significant in reducing the risk of NSCL/P (OR = 0.31, *p* = 0.001). Genetic variation in *FGF1* is an important risk marker of NSCL/P in the Polish population, which cannot be stated for the polymorphisms in the *IRF6* gene.

## 1 Introduction

Annually, more than 8 million children are born worldwide with various birth defects, 17% of which affect the facial part of the skull [1]. Orofacial clefts (OFCs) are the most common congenital anomaly affecting the facial part of the skull and the second most common birth defect in newborn children overall (after phimosis). The most common ORFs are non-syndromic cleft lip with or without cleft palate (NSCL/P), which occur with an average prevalence of 1 in 700 live births [2], and affect 135,000 newborns worldwide [3]. The prevalence of NSCL/P varies among ethnic groups and depends on the geographic origin. Infants with congenital malformations are born significantly more often in parts of South America and Asia, while the fewest are born in Israel, South Africa, and Southern Europe [4]. The cleft palate only (CPO) phenotype occurs less frequently, with a worldwide average of 1–25 in 10,000 children [5]. A higher incidence of isolated cleft palate is reported in Canada and Northern Europe, and significantly lower in parts of Latin America and South Africa [4].

The medical care of children with NSCL/P requires a comprehensive approach. The defect impairs many aspects of daily life, including eating, speech, and due to facial appearance, social interaction with peers [6]. The etiology of ORFs is quite complex, involving both genetic and environmental factors [7]. Many studies indicate that the genetic component has a very strong influence on the development of facial birth defects. The risk of NSCL/P is three times higher in siblings than in the general population and is 25–45% for monozygotic twins compared to 3–6% for heterozygotic twins. Moreover, the risk of birth defects for first-degree relatives is estimated at 4%; for second-degree relatives, 0.67%; and for third-degree relatives, 0.3% [8]. However, the lack of complete coincidence of the occurrence of NSCL/P in monozygotic twins shows that environmental factors also play important roles in the etiology of clefts [9].

Although the last few decades have seen tremendous progress in understanding the genetic basis of syndromic birth defects [10], the identification of specific genetic variants involved in the onset of NSCL/P has progressed at a slower pace, primarily due to the presence of a significant environmental component [2]. Numerous studies have shown that several genes are related to craniofacial deformities [11,12,13,14].

In recent years, advances in research techniques have accelerated the identification of genes and their polymorphisms correlating with NSCL/P occurrence through the use of genome-wide association studies (GWAS) and whole genome sequencing. Numerous studies have shown that a gene whose polymorphisms may be associated with NSCL/P is the *IRF6* gene, mutations of which are responsible for van der Woude syndrome (VWS) [12,15,16]. Loss-of-function mutations in the *IRF6* gene are responsible for approximately 70% of VWS syndrome cases (VWS1, MIM#119300) [17]. Multiple studies have indicated that polymorphisms of the *IRF6* gene could be an important factor in the etiology of non-syndromic malformations [18,19,20]. Genes coding for fibroblast growth factors and their receptors, for instance, *FGF1*, are considered excellent candidate genes. Their proteins play important roles in craniofacial growth and their polymorphisms may influence the abnormal development of palatal and connective tissue structures [21].

The purpose of the present study was to replicate the association between four single nucleotide polymorphisms (SNPs; rs2013162, rs642961, rs2235375, and rs34010) in the IRF6 and the FGF1 gene and NSCL/P in Polish children. Therefore, we compared the frequencies of the four SNPs between children with and without NSCL/P.

## 2 Materials and methods

### 2.1 Study population

We studied unselected children with NSCL/P and healthy controls. The study included patients being treated for orthodontic conditions at the Pomeranian Medical University in Szczecin or the Wroclaw Medical University Department of Dentofacial Orthopeadics and Orthodontics.

In the NSCL/P group (*n* = 209), clinical diagnostics of existing congenital defects and differential diagnostics for monogenic syndromes related to NSCL/P were based on medical and anamnesis history followed by clinical examination. The type of cleft was assessed according to the World Health Organization classification – International Statistical Classification of Diseases and Related Health Problems – ICD 10; Congenital malformations, deformations, and chromosomal abnormalities section (Q35–Q37) [22].

In the control group, 418 unsanctioned children (average age: 14.0 + 10.2 years) were recruited and their genetic material was stored in the Szczecin Department of Genetics and Pathology’s biobank. Patients with NSCL/P and the control group were matched on age and geography. A total of 180 children were enrolled from Szczecin and 238 were enrolled from Wroclaw.

### 2.2 Sample preparation

In the group of children with NSCL/P, 2 ml of saliva samples were collected from each subject using Oragene collection kits (DNA Genotek Inc., Canada). Subjects were asked not to consume any solid food for 30 min before the collection of the biological material. Samples were stored in a dry place, protected from light, at room temperature. DNA was isolated using automatic Chemagen sets. The DNA extracted from the samples was stored in a freezer at −20°C. In the control group, DNA from the umbilical cord blood was extracted using the standard method described by Lahiri and Schnabel [23].

### 2.3 Genotyping

Genotyping of rs2013162, rs642961, rs2235373, and rs34010 was performed using the real-time PCR-based TaqMan technique with LightCycler 480 II (Roche Diagnostics). The mixture (5 µl total) consisted of 2.5 µl of LightCycler 480 Probes Master Mix (Roche Diagnostics), 0.0625 µl of each SNP TaqMan Genotyping Assay × 40 (Applied Biosystems), 1 µl of DNA (25 ng/µl), and 1.4375 µl of deionized water (Roche Diagnostics). On each plate, four negative controls without DNA were included to monitor potential contamination. Primers used for the detection of gene polymorphisms were as follows:

1. rs2013162 forward: CACCTGCGAGCTTGTGTATC; reverse: CCATCATCCCCACTCACCAT,
2. rs642961 forward: GCTTTGGATTGTTAATCTTACCCAAAGG; reverse: CTTCCCACCTCCAGGACAGGCAGATG,
3. rs2235373 forward: AAGTAAGTGAGACTTTATCTTTC; reverse: TCCCTGGTGACTCATGGGCT, and
4. rs34010 forward: GGCCTCTACCCACTAGATGCCAGTAG; reverse: CTGCTTTCATTATCAACCTGCACAGG.

### 2.4 Statistical analysis

A logistic regression test used for nonlinear analysis was used for statistical analysis to describe the effect of independent variables on the dichotomous dependent variable. The odds ratio (OR) with a 95% confidence interval (95% CI) was calculated to estimate the risk of a craniofacial cleft defect. The most common genotype was used as a reference. For each genetic variation, the risk carried by the occurrence of the given genotypes on the occurrence of the birth defect was evaluated with respect to the control group. The significance of individual logistic regression coefficients was evaluated using Wald’s test. The OFC risk was assessed for each genotype. The significance of particular logistic regression rates was assessed using Wald’s test. Statistica 10.0 (StatSoft, Tulsa OK, USA) and R 3.0.2 (The R Foundation for Statistical Computing) were used for statistical analysis. *P*-values of less than 0.05 were considered significant.

Biobank materials obtained from umbilical cord blood were used as the control group, and deposited in the Department of Genetics and Pathomorphology of Pomeranian Medical University in Szczecin. In the control group, individuals were selected based on their age and region of birth.


**Ethical approval:** The Bioethics Committee of Pomeranian Medical University in Szczecin approved the research protocol as complying with GCP – Good Clinical Practice (KB-0012/77/10). Oncology biobank project approved by PAM Ethics Committee (BN-001/174/05 dated 11. 10. 2005). Informed consent was obtained from all patients or their legal guardians before participating in the study.

## 3 Results

The study group consisted of 209 individuals with NSCL/P (age range, 4–30 years; mean age, 17.4 ± 13.6 years). The control group consisted of 418 subjects matched for age and birthplace. Relatives in the ascending line up to the second generation in both groups were Polish. The NSCL/P group consisted of 91 women (43.5%) and 118 men (56.5%). In 113 cases (54.1%), unilateral cleft of the lip and the hard palate was observed; in 45 cases (21.5%), – bilateral cleft of the lip and hard palate; in 32 cases (15.3%), CPO; and in 19 cases (9.1%), isolated cleft lip. The characteristics of the NSCL/P patients are presented in Table 1.

**Table 1:** Characteristics of patients with NSCL/P [24]

		No. and proportion of individuals
Sex	Female	91 (43.5%)
	Male	118 (56.5%)
Cleft type	Unilateral cleft of the lip and the hard palate	113 (54.1%)
	Bilateral cleft of the lip and the hard palate	45 (21.5%)
	CPO	32 (15.3%)
	Isolated cleft lip	19 (9.1%)

Table 2 summarizes the results obtained for the IRF6 gene. Statistical analysis showed that the rs2013162 polymorphism did not significantly predict the risk of the cleft defect (*p* = 0.455). The OR for genotypes AA and AC were, respectively, 1.16 (0.66–2.07) with a significance level of *p* = 0.604 and 0.83 (0.56–1.25) with a significance level of *p* = 0.382. A similarly statistically insignificant result was obtained for the rs642961 polymorphism (*p* = 0.189). In this case, the OR was 0.84 (0.34–2.08) for the AA genotype, with a significance level of *p* = 0.709, and 1.41 (0.95–2.07) for the AG genotype, with a significance level of *p* = 0.085. Also, the analysis of the rs2235373 polymorphism showed no significant (*p* = 0.716) change in the risk of the OR of congenital craniofacial malformations with OR = 0.79 (0.24–2.68), *p* = 0.71 (for the AA variant) and OR = 0.85 (0.55–1.31), *p* = 0.453 (for the AG variant). On the other hand, the presence of the rs34010 polymorphism statistically significant affects the risk of developing a cleft defect (*p* < 0.001, Table 3). A reduced risk (OR = 0.31 (0.18–0.54)) is carried out by the presence of the AA genotype (*p* < 0.001) and the AC genotype, but in this case this was not confirmed by statistical significance (*p* = 0.219).

**Table 2:** NSCL/P risk and polymorphisms in the *IRF6* gene (namely, rs2013162, rs642961, rs2235373)

Genotype	OR (95% CI)	*P* (Wald’s test)	*P* (LR-test)
**rs2013162 ref. = CC**			0.455
AA	1.16 (0.66–2.07)	0.604	
AC	0.83 (0.56–1.25)	0.382	
**rs642961 ref. = GG**			0.189
AA	0.84 (0.34–2.08)	0.709	
AG	1.41 (0.95–2.07)	0.085	
**rs2235373 ref. = GG**			0.716
AA	0.79 (0.24–2.68)	0.71	
AG	0.85 (0.55–1.31)	0.453	

**Table 3:** NSCL/P risk and rs34010 polymorphism in the *FGF1* gene

Genotype	OR (95% CI)	*P* (Wald’s test)	*P* (LR-test)
**rs34010 ref. = CC**			<0.001
AA	0.31 (0.18–0.54)	<0.001	
AC	0.77 (0.51–1.17)	0.219	

**Figure 1:**
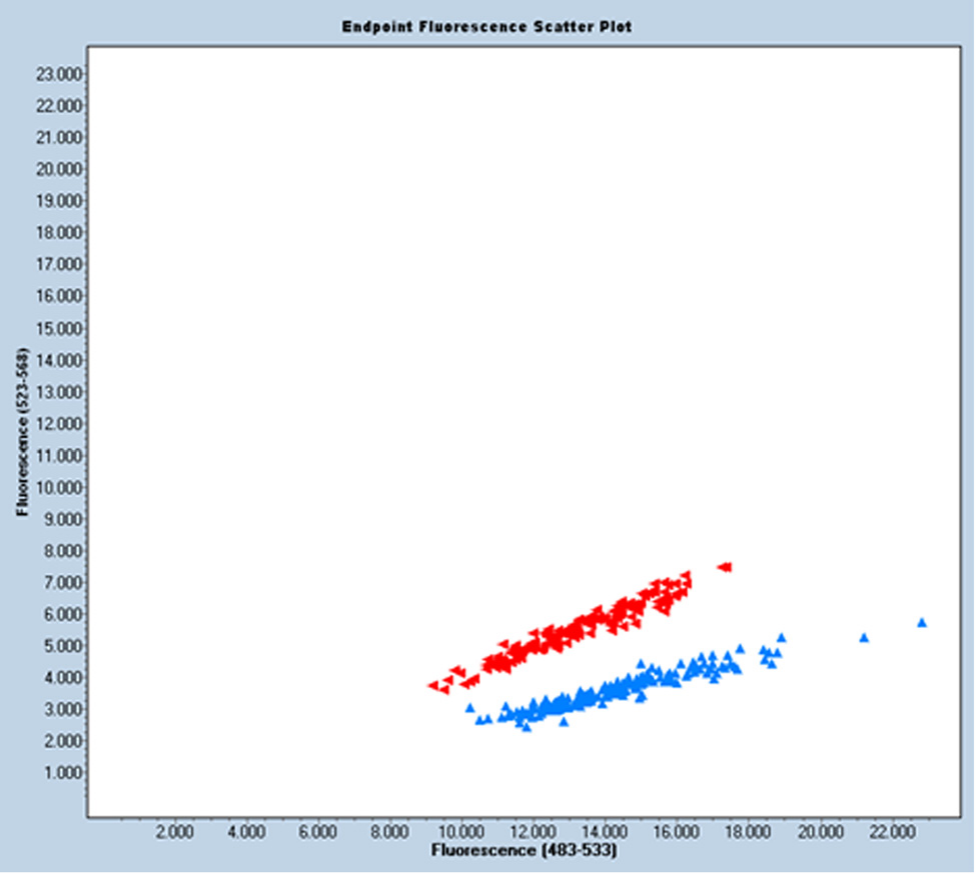
Graphical distribution of genotypes for rs34010.

The distribution of genotypes for rs34010 is shown in Figure 1. The presented result of the fluorescence analysis of the sample was performed using a LightCycler 480II. The blue color represents the AC allele (stained with VIC dye); the green color, if present, would represent the AA allele (stained with FAM dye); and the red color represents both alleles.

## 4 Discussion

In the present study, we found an association between the presence of SNP rs34010 within *FGF1* and the risk of NSCL/P in a Polish population. The same results were obtained in a study conducted on Latvian, Lithuanian, and Estonian populations. It was shown that the occurrence of a rarer allele of the rs34010 polymorphism in the *FGF1* gene is associated with a reduced incidence of the cleft defect. A particularly strong association with a decreased risk (OR = 0.689; 95% CI, 0.559–0.849; *p* = 4.56 × 10−4) was found for the protective rs250092/rs34010 GT haplotype, within the aforementioned gene [25].

The *FGF* gene family signaling pathway is responsible for the regulation and proper execution of many developmental processes as early as embryogenesis. It plays an important role in the formation of head structures, particularly the craniofacial region and palate development. Conducted studies in the *FGF* gene family have shown an overrepresentation of missense mutations in patients with cleft palates compared with healthy individuals. It has been shown that a group of missense and nonsense mutations (M369I, E467K, R609X, D138N, R84S, V329I, D73H *de novo*, S59F, K172) can account for up to 5% of NSCL/P cases. Taken together with the results for *IRF6* −12%, *FOXE1, GLI2, MSX2, SKI*, and SPRY2 −6%, and *MSX1* −2% [11,12,26], these genes may account for more than 25% of NSCL/P and represent a significant predictor of facial clefts [27]. The missense and nonsense *FGFR1* mutations indicate problems with reduced penetrance when identifying mutations in ORFs. The R609X mutation was found in a father and his daughter. The M369I mutation correlates with the cleft phenotype in the family, but there were family members without a birth defect with this mutation, who may be examples of reduced penetrance. Some apparently nonpenetrating individuals in ORF families show other phenotypic features, such as discontinuity of the orbicularis oris muscle [28,29].

Human FGFs control a wide range of biological functions, and their biological activity is mediated by seven major FGF receptor tyrosine kinases encoded by four genes (*FGFR1*-*4*). In the presence of heparan sulfate proteoglycans, two FGFs bind to two FGFRs, inducing receptor dimerization and allowing intracellular tyrosine kinase domains to phosphorylate and activate [30]. As a result of impaired FGF signaling, craniofacial syndromes, craniofacial dysplasia, and Kallmann syndrome may result. The S252W and P253R mutations in the *FGFR2* gene are responsible for almost all cases of Apert syndrome. The cleft type is associated with the S252W mutation in 59% of patients, with the P253R mutation in 17% of patients. Cleft palate is present in 44% of Apert syndrome cases [31,32,33]. Autosomal-dominant Kallmann syndrome, whose main features are anosmia and hypogonadism, is caused by loss-of-function mutations in *FGFR1*, and 5–10% of these patients have cleft [34,35,36].

In this study, we found no association between the presence of *IRF6* gene SNPs and the occurrence of NSCL/P. Interferon regulatory factor (IRF6) plays an important role during embryonic development. This factor is responsible for immune system function and wound healing [37]. Unlike the other interferon regulatory factors, *IRF6* is also crucial during fetal cranial growth and the development of ectodermal structures [38]. Lack of *IRF6* expression may implicate birth defects such as VWS and popliteal pterygium syndrome [39]. These defects are characterized by the presence of a cleft palate and abnormalities of the teeth (20–40%) and skin and mucous membranes [40].

For the rs2013162 SNP located in the *IRF6* gene in the present study, ORs were 1.16 for the AA genotype and 0.83 for the AC genotype, but these results were not statistically significant. Similarly, a statistically insignificant result was obtained by Huang et al. subjecting a Chinese population [41]. But these results are significantly different from those conducted previously on Italian, American, and Belgian populations [42,43,44], in which a strong association between the occurrence of rs2013162 polymorphism and the occurrence of isolated cleft facial defects was demonstrated, and by Park et al. [45] on the Chinese population. Also in each of the above-mentioned studies, in the haplotype analysis, it was noted that the C allele was present in all individuals affected by cleft lip or palate.

The literature data suggest a correlation of rs642961 with the occurrence of craniofacial cleft defects. The results of the studies present a contradictory view. The first study was conducted on the Brazilian population, but the researchers chose a small size of the control group, which, with MAF *A* = 0.017 (characterizes this variation), may not be a representative result for the entire population. In their study, 228 cleft patients and 126 healthy subjects were examined [46]. Another publication in which the results indicate that there is no association between rs642961 and the occurrence of isolated facial cleft defects is based on a study of a Honduran population [19]. However, other publications describe examples of positive correlations with the occurrence of cleft defects. A paper describing a Brazilian population presents a correlation between the polymorphisms and the occurrence of cleft, although only in a group of individuals with isolated cleft palate [47]. Researchers from China, in addition to finding a relationship between this genetic variation and the occurrence of congenital cleft defects, demonstrated that rs642961 can alter the expression of the *IRF6* gene *in vivo*. They have surgically removed a small section of the lip skin with tissue adjacent to the region of the cleft lip present. They have indicated that the genotypes of this polymorphism are in correlation with different expression levels of *IRF6* gene receptors [48]. Interestingly, studies conducted on a Polish group of patients show the result of a positive correlation with the occurrence of isolated facial cleft defects, which is statistically significant. The reported OR coefficient was 1.63, with a significance level of *p* = 0.05 [49]. Also, a high OR coefficient (1.79) at the retained level of significance is reported in the results of their work by Birnbaum et al. whose study was based on the examination of genetic material collected from 460 subjects with isolated cleft lips with or without cleft palate and from 952 control subjects, representatives of the Caucasian population [50].

An interesting finding was made by Murdoch et al., who examined the absence of tooth buds and palate type in association with a given genotype. As a result, it has been found that the first palatal folds on the right side were larger than on the left side in the group of individuals with the rs642961 polymorphism, although *p* = 0.06, indicating the need for further research. No correlation was found concerning hypodontia [51].

In the present study, the results obtained for the *IRF6* rs2235373 gene polymorphism were not found to be statistically significant. In the literature, evidence showing a sevenfold increased predisposition to congenital malformations correlated with the co-occurrence of haplotypes: GC rs2235373-rs2235371 (V274I) and AAG rs599021-rs2235373-rs595918 can be found [45]. A study including a Norwegian population (377 patients with isolated cleft lip with or without cleft palate, 196 patients with isolated cleft palate, and 763 healthy subjects) showed a reduced risk (OR = 0.38, *p* < 0.001) of isolated cleft lip with or without cleft palate in the presence of the rs2235371 (V274I) genotype, also located in the *IRF6* gene. However, no association between the aforementioned polymorphism and the presence of an isolated cleft palate was demonstrated [52].

Studies in the Han population have shown that the risk of having a child with an isolated cleft lip with or without a cleft palate is increased for mothers who were taking medication or were exposed to passive smoking during the first trimester of pregnancy. Folic acid supplementation at the recommended dose had a protective effect on fetal development. The concomitant presence of the TT genotype of the rs2235373 polymorphism and a history of previous miscarriage increase the risk of an isolated cleft defect more than sixfold (OR = 6.7) [53]. Studies demonstrate the interaction of *IRF6* and *TGFA* gene polymorphisms and an increased risk of the craniofacial cleft with their simultaneous presence [54].

In the Polish population, Mostowska et al. analyzed 18 polymorphisms of *FOXE1, IRF6, MSX1, PAX9, TBX10, FGF10, FGFR1, TGFa, TGFb3,* and *SUMO1*, and chromosomal region 8q24 in a group of 175 children with NSCL/P and a matched control group. A strongly significant association with NSCL/P risk was observed for rs642961 in *IRF6* (OR [AG + AA vs GG] = 1.635, 95% CI 1.153–2.319, *p* = 0.005) [49]. Mostowska et al. also examined variants located in chromosomal regions 1p22.1, 10q25.3, 17q22, and 20q12 in a group of 206 individuals with NSCL/P and a matched control group of 446 individuals. As a result, rs227731 (located on 17q22) increased the risk of NSCL/P in the analysis in the dominant model (OR = 1.732, 95% CI 0.184–2.253, *p* = 0.0044). The lack of convergence of results in the Mostowska et al. [55] study may reflect differences in the geographic origin of cases compared with our study.

Reports to date on the predisposition of genetic variations to isolated cleft defects are usually based on single studies and are not in accordance with different research centers. Often, these studies are based on populations that are ethnically distinct from the Polish ones. Furthermore, there are no studies that clearly state that the predisposition for isolated cleft defects is based on a single gene. It is difficult to find papers that unambiguously point in the direction of research aimed at creating a probabilistic model of the etiology of ORFs that could be established [56].

### 4.1 Limitations

There has been extensive research on the etiology of NSCL/P but the results have been inconsistent. A study based on the association between SNPs and the presence of different phenotypes may have limited power to detect relevant complex inheritance patterns (e.g., synergistic involvement of different polymorphisms or interactions between environmental factors). In fact, ORs tend to be low or moderate when we examine the association between single genes and the risk of a complex trait that is likely to be regulated by a large number of genes. As a result, phenotypes result from a combination of genes and environmental factors. The residual genetic risk of nonsyndromic craniofacial clefts must therefore be explained by gene–environment interactions.

At this point, it is also worth noting that the conducted case–control studies are less adept at showing a causal relationship than cohort studies. They are also more prone to bias. The same hypothesis could also be studied in population-based cohort studies. Case–control studies do not require a long follow-up period and are easier to conduct. This design is especially useful for rare diseases but not for rare causes.

## 5 Conclusions

This is the first study to investigate the association between *IRF6* and *FGF1* genotypes and NSCL/P in the Polish population. Our findings suggest that rs34010 in the *FGF1* gene is associated with a decreased risk of NSCL/P, while rs2013162, rs642961, and rs2235373 in the *IRF6* gene require further study due to lack of statistical significance. This study contributes to our understanding of genetic factors related to NSCL/P; however, further investigation in a larger population is warranted.
